# Evolution of resistance and tolerance to herbivores: testing the trade-off hypothesis

**DOI:** 10.7717/peerj.789

**Published:** 2015-03-03

**Authors:** Eunice Kariñho-Betancourt, Juan Núñez-Farfán

**Affiliations:** Laboratorio de Genética Ecológica y Evolución, Departamento de Ecología Evolutiva, Instituto de Ecología, Universidad Nacional Autónoma de México, Circuito Exterior, Ciudad Universitaria, México, DF, Mexico

**Keywords:** Genetic variation, Herbivory, Resistance, Leaf trichomes, Natural selection, Plant defense, Trade-offs, Tolerance

## Abstract

**Background**. To cope with their natural enemies, plants rely on resistance and tolerance as defensive strategies. Evolution of these strategies among natural population can be constrained by the absence of genetic variation or because of the antagonistic genetic correlation (trade-off) between them. Also, since plant defenses are integrated by several traits, it has been suggested that trade-offs might occur between specific defense traits.

**Methodology/Principal Findings.** We experimentally assessed (1) the presence of genetic variance in tolerance, total resistance, and leaf trichome density as specific defense trait, (2) the extent of natural selection acting on plant defenses, and (3) the relationship between total resistance and leaf trichome density with tolerance to herbivory in the annual herb *Datura stramonium*. Full-sib families of *D. stramonium* were either exposed to natural herbivores (control) or protected from them by a systemic insecticide. We detected genetic variance for leaf trichome density, and directional selection acting on this character. However, we did not detect a negative significant correlation between tolerance and total resistance, or between tolerance and leaf trichome density. We argue that low levels of leaf damage by herbivores precluded the detection of a negative genetic correlation between plant defense strategies.

**Conclusions/Significance.** This study provides empirical evidence of the independent evolution of plant defense strategies, and a defensive role of leaf trichomes. The pattern of selection should favor individuals with high trichomes density. Also, because leaf trichome density reduces damage by herbivores and possess genetic variance in the studied population, its evolution is not constrained.

## Introduction

Plants rely on resistance and tolerance to avoid the negative fitness effects of damage by herbivores or pathogens (Simms & Triplett, 1994; Fineblum & Rausher, 1995; Strauss & Agrawal, 1999; Núñez-Farfán, Fornoni & Valverde, 2007). Resistant plants prevent or reduce the amount of damage through chemical secondary metabolites and physical defenses (Ehrlich & Raven, 1964; Levin, 1973; Berenbaum, Zangerl & Nitao, 1986; Wink, 2003). Tolerant plants can lessen the negative impact of herbivore damage on fitness, once it has occurred (Rausher, 1992b; Stowe et al., 2000). Unlike resistance, tolerance does not prevent herbivory but maintains fitness by eliciting compensatory physiological plant responses after damage by herbivores.

Natural selection for increased resistance to herbivores has been detected in different plant species as well as for plant traits associated to resistance (Berenbaum, Zangerl & Nitao, 1986; Simms & Rausher, 1989; Mauricio & Rausher, 1997; Shonle & Bergelson, 2000). However, investment in plant defense is thought to involve fitness costs such that optimal defense does not necessarily imply maximal investment (Fagerström, Larsson & Tenow, 1987; Simms & Rausher, 1987). Thus, potential trade-offs between different defensive traits might arise (see Mauricio, 1998). Furthermore, if fitness costs of herbivory can be reduced by tolerance, selection on resistance traits would be relaxed, nil, or even selected against if leaf damage could be partially or completely compensated by tolerance (Abrahamson & Weis, 1997; Fineblum & Rausher, 1995; Mauricio, Rausher & Burdick, 1997). Hence, the simultaneous investment in tolerance and resistance may imply a greater total cost than the possession of only one pure strategy (van der Meijeden, Wijn & Verkaar, 1988; Herms & Mattson, 1992; Fineblum & Rausher, 1995; Mauricio, Rausher & Burdick, 1997). However, if the fitness benefit of investment in tolerance and resistance is higher than its cost, the evolution of a mixed defense strategy is a possible outcome (Fornoni et al., 2004a; Carmona & Fornoni, 2013).

Nevertheless, evidence of a trade-off between plant defensive strategies is scarce, and may depend on the sort of traits involved in the defensive response. A review of literature indicates little support for a negative genetic correlation between tolerance and resistance across different plant species, and suggests that a fruitful approach is to assess the relationship between tolerance and specific plant resistance traits rather that the correlation between tolerance with total resistance (Leimu & Koricheva, 2006). Thus, this study aimed to determine if total resistance and a component of it (leaf trichomes) are genetically correlated with plant tolerance. We carried out an experiment to expose maternal half-sib families of the annual plant *Datura stramonium* to their natural herbivores in order to (1) assess genetic variation in plant tolerance, total resistance, and leaf trichome density, and (2) measure selection on trichome density and resistance to herbivores. Finally, (3) we estimated the genetic correlation between tolerance and total resistance and leaf trichomes.

## Materials and Methods

### Study system

*Datura stramonium* L. (Solanaceae) is an annual herbaceous plant native to Mexico, but widely distributed worldwide. It is commonly found as ruderal in disturbed habitats (Weaver & Warwick, 1984; Núñez-Farfán & Dirzo, 1994; Shonle & Bergelson, 2000). In central Mexico, its leaves are consumed by specialist herbivorous insects (i.e., those that feed upon a restricted group of related plants), such as the leaf beetles *Epitirx parvula* and *Lema trilineata* (Coleoptera: Chrysomelidae) (Núñez-Farfán & Dirzo, 1994; Castillo et al., 2013), and generalist herbivores (i.e., that feed upon several unrelated plant species), such as the grasshopper *Sphenarium purpurascens* (Orthoptera: Pyrgomorphidae). Also, the specialist weevil *Trichobaris soror* (Coleoptera: Curculionidae) is a seed-predator of *Datura stramonium* in populations of central Mexico (Núñez-Farfán & Dirzo, 1994; Núñez-Farfán, Cabrales-Vargas & Dirzo, 1996). Previous studies in *D. stramonium* have shown that damage caused by these insects reduces plant fitness (Núñez-Farfán & Dirzo, 1994; Valverde, Fornoni & Núñez-Farfán, 2001; Fornoni, Valverde & Núñez-Farfán, 2004b), and that tropane alkaloids and leaf trichomes confer resistance against its natural herbivores (Shonle, 1999; Valverde, Fornoni & Núñez-Farfán, 2001; Kariñho-Betancourt, 2009; Castillo et al., 2014). Likewise, variation among-population in such defensive traits (alkaloids and leaf trichomes) is associated with the composition of the herbivore community (Valverde, Fornoni & Núñez-Farfán, 2003; Fornoni, Valverde & Núñez-Farfán, 2003; Castillo et al., 2013; Castillo et al., 2014).

Based on previous studies with this species, we selected the Ticuman population of *D. stramonium* because genetic variance for resistance and tolerance was detected (Fornoni, Valverde & Núñez-Farfán, 2003), and leaf trichome density correlates with leaf damage by herbivores (Valverde, Fornoni & Núñez-Farfán, 2001). The vegetation in the Ticuman locality (18°47′N and 99°06′W) is a tropical dry forest at 990 m.a.s.l., with an average annual precipitation and temperature of 954.4 mm and 24 °C, respectively. Plants in this locality receive low levels of average damage by herbivores 10.9 ± 4.0%; mean ± SE; (Valverde, Fornoni & Núñez-Farfán, 2001; Fornoni, Valverde & Núñez-Farfán, 2003), compared to other populations that share the same herbivores (Valverde, Fornoni & Núñez-Farfán, 2001).

### Experimental Design

Experimental plants were obtained in a greenhouse by sowing seeds of each of 28 maternal half-sib families (natural progenies; Lawrence, 1982). Once the first two true leaves appeared, plants were transplanted to an experimental plot in Ticuman under a randomized block design, and watered regularly each week.

To assess the pattern of selection on resistance characters in the presence and absence of natural herbivores (*e.gr.,* Mauricio & Rausher, 1997), and evaluate the cost of defensive traits, we selected a sample of 16 families and divided the progeny of each family in two groups of insecticide treatment (control and treated). We used a systemic carbofuran insecticide (FURADAN^®^; FMC Corporation, Philadelphia, Pennsylvania, USA). Two weeks after transplanting, we watered the experimental plants with 500 ml of a solution containing the insecticide. The same volume of water was supplied to control plants.

Plants of each family were measured for (1) plant height, (2) stem diameter, (3) number of branches, (4) number of flowers, (5) number of fruits, (6) total seeds, (7) leaf damage by herbivores, and (8) leaf trichome density.

### Resistance to herbivores

To estimate total plant resistance, *R_i_*, we randomly choose a sample (*n*) of 20 leaves per plant *i*. For each leaf we measured total (*A_T_*) and damaged area (*A_D_*) by using a leaf area meter (Winfolia; Regent Instruments Inc., Québec, Canada). Thus, relative resistance to herbivores of plant *i*(*R_i_*) is related to the proportion of leaf area damaged (*D_i_*) as: ${R}_{i}=1-{D}_{i}=1-\left(\frac{1}{n}\sum _{i=1}^{n}\frac{{A}_{D}}{{A}_{T}}\right)$, (Núñez-Farfán & Dirzo, 1994). This estimate of resistance to herbivores (*R_i_*) has been broadly related as a measure of total resistance (see Leimu & Koricheva, 2006).

To measure the plant’s leaf trichome density, we counted the number of trichomes in three areas of 1.7 mm^2^ in the abaxial side of each leaf (at the bottom, right and left edges of the leaf) using a dissection microscope (Valverde, Fornoni & Núñez-Farfán, 2001).

### Reproductive output

We counted the total number of fruits and seeds produced by each plant (*W_i_*) in order to obtain an estimator of maternal plant fitness. Following Lande & Arnold (1983) we defined relative fitness (*w_i_*) as, ${W}_{1}=\frac{{W}_{i}}{\overline{W}}$ where $\overline{W}$ is the average number of fruits or seeds per plant in the population. Since fitness estimated either as total fruits and total seed number were positively correlated (*r* = 0.9, *P* < 0.0001), we used the estimate based on seed number for subsequent statistical analyses.

### Plant tolerance

Using plants exposed to herbivores, we estimated tolerance of each family as the slope (*β_i_*) of a linear regression between individual relative fitness (*w_i_*) *versus* relative damage received (*D_i_*) by herbivores (Mauricio, Rausher & Burdick, 1997; Fornoni, Valverde & Núñez-Farfán, 2003). Since tolerance benefits are expressed in the presence of damage, plants treated with insecticide were not included in this analysis.

### Data analysis

#### Genetic variance and heritability

In order to estimate additive genetic variance, an ANOVA for each character was carried out with the family term as a random variable and the block as the fixed effect. Broad-sense heritability (${h}_{B}^{2}$) was estimated as the ratio between twice the family component of variance (${\sigma }_{f}^{2}$) divided by the total phenotypic variance (${\sigma }_{p}^{2}$), since resemblance among members of a family (i.e., covariance) contains one half of additive genetic variance (Falconer & MacKay, 1995). Genetic correlation among characters were estimated as the correlation between family means (Via, 1984).

Genetic variance in tolerance was assessed by means of an ANCOVA of fitness as a function of the family, the relative damage by herbivores (the covariate), and the interaction family × relative damage. A significant family × relative damage suggests genetic variance in the reaction norms of fitness in relation to damage by herbivores (Fornoni, Valverde & Núñez-Farfán, 2003).

#### Correlation between leaf damage and resistance traits

In order to assess the relationship of leaf trichome density and damage, i.e., the defensive role of leaf trichomes, we performed a correlation analysis with plants exposed to herbivores. The analysis was conducted using the individual values of leaf trichome density, total resistance, relative leaf damage, and relative plant fitness.

#### Cost analysis

The cost of resistance attributes was estimated using plants that received the insecticide application by performing a linear regression of (1) total resistance and relative fitness (*w_i_*), and (2) leaf trichome density and relative fitness (*w_i_*). A negative slope indicates costs for the defensive traits.

#### Selection analysis

Natural selection on plant resistance attributes (total resistance and leaf trichomes) was estimated by a partial linear regression of fitness to detect (1) directional selection (*β_i_*), and/or (2) non-linear selection (*γ_ij_*) by means of partial quadratic regression of fitness as a function of the quadratic values of characters (Lande & Arnold, 1983). Selection analyses were performed on phenotypic and breeding values (Rausher, 1992b; Mauricio & Mojonnier, 1997).

#### Genetic correlation (trade-off) between resistance and tolerance

To assess the genetic correlation between resistance and tolerance, we performed a correlation analysis between the family average values of resistance traits (total resistance and leaf trichome density) and tolerance.

## Results

**Figure 1:**
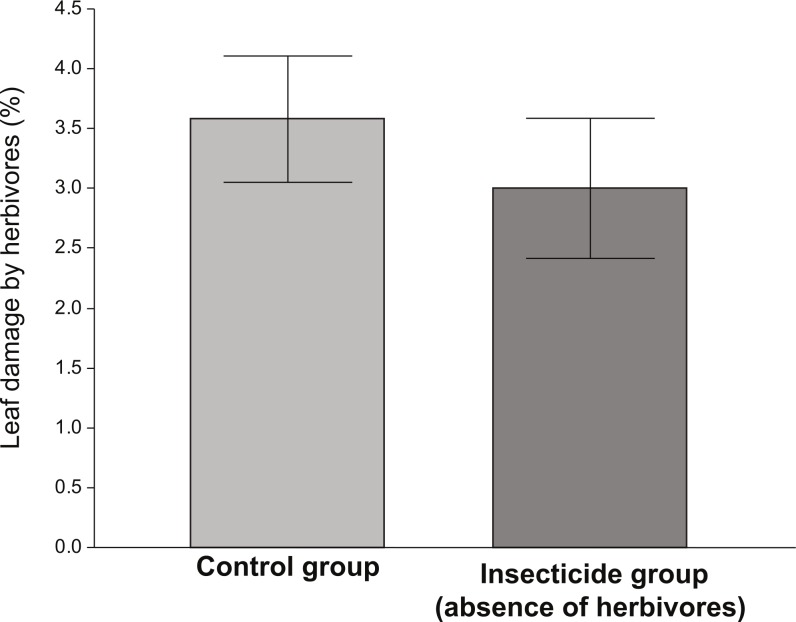
Percentage of leaf damage by herbivory between control and insecticide group.

The amount of damage received by plants exposed to herbivores (control) was significantly higher than that received by plants in the insecticide group (Fig. 1). Although the levels of damage were low in both groups, the ANOVA indicated that plants that received the insecticide application were significantly less damaged (∼15%) than those who did not (*F*_1,135_ = 5.83, *P* = 0.017). However, in spite of the fact that plants performing better in the absence of herbivores (insecticide group), the differences in the average values of vigor, reproductive and resistance traits between the control and the insecticide group were not significant (Table 1).

**Table 1:** Average values (SE) of vigor, reproductive, and defense characters of *Datura stramonium*. The *F* and *P* values are derived from the analysis of variance for the insecticide group (I) and the replicate plants from the control group (C) (herbivores present).

Character	${\bar {x}}_{(C)}$ (SE)	${\bar {x}}_{(I)}$ (SE)	*F*	*P*
Plant height	5.55(0.06)	5.54(0.06)	0.008	0.926
Steam diameter	1.94(0.04)	1.97(0.05)	0.172	0.679
Branch number	1.99(0.09)	1.97(0.09)	0.043	0.836
Flower number	1.94(0.08)	1.86(0.09)	0.402	0.527
Fruit number	1.57(0.1)	1.63(0.1)	0.16	0.69
Seed number	5.85(0.21)	6.24(0.23)	1.477	0.227
Leaf trichome density	3.85(0.06)	3.83(0.07)	0.066	0.798

### Genetic variance

**Figure 2:**
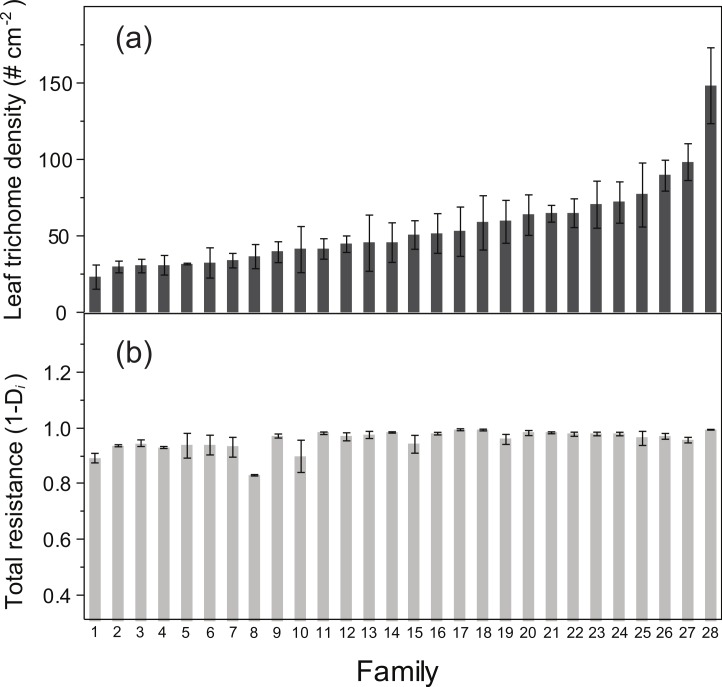
Average values (± SE) of (A) leaf trichome density and (B) total resistance in families of *Datura stramonium*.

ANOVA detected a significant family effect of the number of flowers produced per plant and the two resistance traits estimated: total resistance (*F*_27,125_ = 1.57, *P* = 0.04) and leaf trichome density (*F*_27,125_ = 3.62, *P* < 0.001) (Table 2; Fig. 2). Heritability of leaf trichome number was high (*h*^2^ = 0.641), compared to total resistance (*h*^2^ = 0.259). In contrast, we failed to detect genetic variation of tolerance to damage, since the family × relative damage by herbivores was not significant (*F*_27,125_ = 0.08, n.s.).

**Table 2:** Analysis of variance of reproductive (flowers) and resistance traits (total resistance and leaf trichomes density). The family is the random effect and block is a fixed factor. A significant effect of family (*P* < 0.05) indicates that there is genetic variance. The analysis was performed with plants exposed to herbivores.

Character	Source of variation	d.f.	*F*	*P*
**Flower number**	Family	27	1.94	**0.01**
	Block	2	6.32	**0.002**
	Error	90		
	Total	119		
**Total resistance**	Family	27	1.64	**0.04**
	Block	2	0.81	0.44
	Error	96		
	Total	125		
**Leaf trichome density**	Family	27	3.06	**<0.0001**
	Block	2	4.62	**0.012**
	Error	96		
	Total	125		

**Notes.**

Values in bold indicate *P* < 0.05.

### Correlation between leaf damage and resistance traits

We found a positive phenotypic correlation (individual values) between leaf trichome density, and relative fitness. Also, a negative correlation between leaf damage and leaf trichome density (*r* = − 0.17, *P* = 0.04) was detected. However, only the genetic correlation (family values) between leaf trichome density and relative fitness was significant (*r* = 0.83, *P* < 0.001) (Table 3).

**Table 3:** Phenotypic (above the diagonal), and genetic (below the diagonal) correlations between leaf damage, leaf trichome density, relative fitness (*wi*), and total resistance in *Datura stramonium* plants. The analysis was performed with plants exposed to herbivores.

	Leaf damage (relative)	Leaf trichome density	Relative fitness (wi)	Total resistance
**Leaf damage (relative)**	1.0	−0.179^*^	−0.109	−1.0
**Leaf trichome density**	−0.29	1.0	0.562^***^	0.18^*^
**Relative fitness (*wi*)**	−0.3	0.83^***^	1.0	0.11
**Total resistance**	−1.0	0.29	0.3	1.0

**Notes.**

* *P* < 0.05.

*** *P* < 0.0001.

### Costs

We failed to detect significant costs for any of the resistance attributes. In fact, contrary to expectations, the relationship between leaf trichomes and plant fitness was positive even in the absence of herbivores (*r* = 0.92, *P* = 0.0006).

### Natural selection on resistance and tolerance

**Figure 3:**
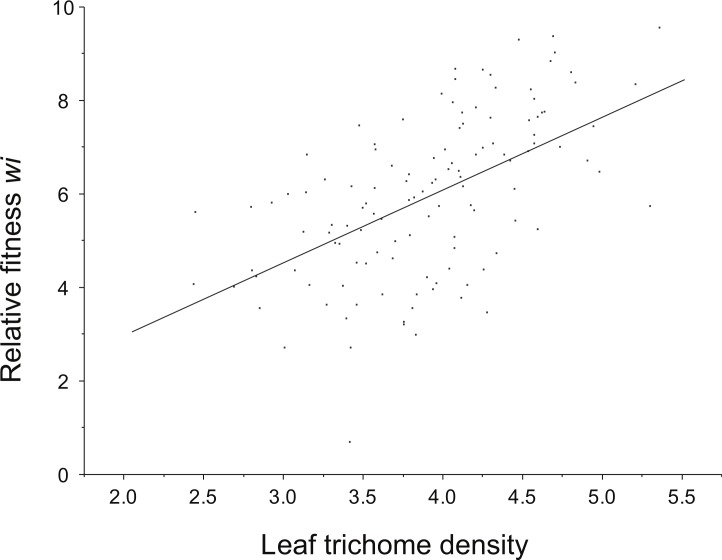
Relationship between leaf trichome density and relative plant fitness in the presence of herbivores.

Positive directional selection was detected on leaf trichome density (resistance component) (Fig. 3). However, directional selection was not significant for total resistance. No non-linear selection (curvilinear) for any of the resistance attributes (leaf trichomes and total resistance) was detected (Table 4). Also, neither directional nor non-linear selection acting on tolerance was found.

**Table 4:** Linear (**β**), and non-linear (**γ**) selection gradients on resistance traits to herbivores for *Datura stramonium* plants growing in Ticuman, Morelos.

Character	**β**_1_	*t*	*P*	ANOVA of the multiple linear regression model	*γ*	*t*	*P*	ANOVA of the multiple quadratic regression model
Total resistance	0.179 (0.145)	1.23	0.22	*F* = 57.27 *P* < 0.0001 *R*^2^ = 0.31	−0.002 (0.061)	−0.04	0.96	*F* = 14.02 *P* < 0.0001 *R*^2^ = 0.31
Leaf trichome density	0.89 (0.117)	7.57	**<0.0001**		0.024 (0.065)	0.37	0.7	

**Notes.**

Values in bold indicate *P* < 0.05.

### Genetic correlation (trade-off) between resistance and tolerance

We did not detect the presence of a trade-off between defensive strategies (resistance and tolerance). The correlation between tolerance and the family averages of the two resistance attributes analyzed was not significant (leaf trichomes density: *r* = − 0.217, *P* = 0.226; total resistance: *r* = 0.155, *P* = 0.431).

## Discussion

Selection to increase leaf trichome was detected in the population of *D. stramonium*. This character was negatively related to damage by herbivores. However, we did not detect selection on total resistance suggesting that individual variation in resistance includes other components besides leaf trichome density (Agrawal, 2011). Although we found evidence of genetic variation in resistance and leaf trichome density, we failed to detect genetic variation for plant tolerance to damage. Hence, we found not support for the trade-off hypothesis between plant resistance and tolerance, or between tolerance and a specific resistance trait (leaf trichome density).

Furthermore, we did not detect fitness costs of leaf trichome density in the absence of herbivores. In fact, the effect of this trait fitness was positive even in the absence of herbivores, suggesting that such resistance trait could be correlated with other traits that were subject to selection (Björkman & Anderson, 1990; Roy, Stanton & Epplely, 1999), or that leaf trichomes may have another function besides defense. Empirical evidence has shown that, in addition to being a mechanical barrier to herbivores (Baur, Blinder & Benz, 1991; Agrawal et al., 2009), nonglandular trichomes (not-producing chemical compounds) can reduce the amount of heat on the leaf surface (Ehleringer, Björkman & Mooney, 1976; Vogelmann, 1993), thus reducing water loss through evapotranspiration. Nevertheless, our results are consistent with a defensive role, even when defense would not be the primary function of leaf trichomes.

**Figure 4:**
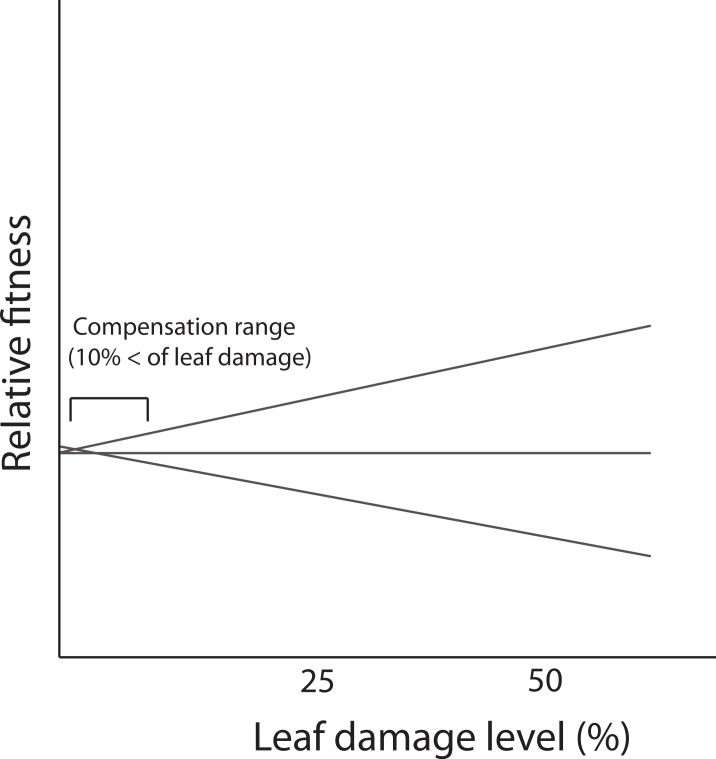
Fitness reaction norms of hypothetical genotypes as a function of damage by herbivores. Genetic variance in reaction norms would be detected when damage is over 10%.

In the present study, we did not detect genetic variance for tolerance contrasting with results reported for the same population by Fornoni, Valverde & Núñez-Farfán (2003). A possible explanation is that the low levels of leaf damage recorded in this study (86% of individuals that received leaf damage, experienced less than 10% of loss) prevented the expression of differences in tolerance i.e., lack of genetic variation. Previous evidence indicates that *D. stramonium* in the same population receives, on average, more damage by herbivores (21.65% ± 0.7; mean ± SE) than the average level recorded in this study. In the Ticuman population, the detection of genetic variance occurred when the damage surpassed 10% of total leaf area (see Fornoni, Valverde & Núñez-Farfán, 2003). Thus, if tolerance is a genotype’s reaction norm of fitness in a damage gradient, differences in the reaction norms (i.e., G × E interaction, implying tolerance) could be detected when damage attains higher values. But at low levels of leaf damage, only part of the reaction norm is apparent in the narrow range of the damage gradient and no differences among genotypes is present (Fig. 4). This fact might possibly preclude the detection of a significant correlation between defensive strategies (or a specific resistance trait). Previous studies have shown how the correlation between resistance and tolerance may vary depending on the biotic environment (e.g., levels of leaf damage due to herbivory). For instance, Fornoni, Valverde & Núñez-Farfán (2003) assessed the correlation between defensive strategies by conducting a reciprocal transplants experiment between two natural populations of *D. stramonium*. They found that the detection of a trade-off between total resistance and tolerance occurred only for native plants growing in the population with the highest levels of leaf damage (i.e., Ticuman). In contrast, in the population where the average level of leaf damage was lower (i.e., Santo Domingo) no trade-off was detected, suggesting that the amount of leaf damage may restrict the detection of a significant correlation between plant defenses.

The expression of a negative correlation could also be restricted by patterns of resource acquisition and allocation to defense. Theoretically, if variation in resource allocation is greater than variation in acquisition (i.e., increasing defense costs), a negative correlation between defenses is a possible outcome (Houle, 1991; Mole, 1994). Allocation patterns depend mainly on the frequency and intensity of herbivory during the plant’s life (Basey, Jenkins & Busher, 1988; Boucher, 1985; Langenheim & Stubblebine, 1983). The low levels of leaf damage detected in this study may indicate (besides of resistance traits acting on plant consumers) a low abundance of herbivores. This factor could reduce the variance of resource allocation to defense. Consequently, defense costs could be diminished, and plants could simultaneously allocate resources to different *classes* of defensive traits, i.e., a trade-off between defensive strategies would not be favored.

On the other hand, the trade-off hypothesis between defensive strategies is based on the assumption of redundancy. However, the defensive role of plant defense-related traits (strategies) would depend on the identity and diversity of herbivores attacking the plants. Previous studies on *D. stramonium* have shown how different herbivores could modify the selection pattern on resistance traits (Shonle & Bergelson, 2000; Lankau, 2007), or defense strategies (Carmona & Fornoni, 2013), and how geographic variation of the herbivore community is related to variation in the selective patterns exerted by plant consumers on chemical and physical resistance traits.

## Conclusions

Altogether, our results suggest that resistance could evolve independently from plant tolerance in the Ticuman population. Leaf trichome density is a heritable resistance component and thus it has no restrictions on evolving, and the pattern of selection should favor those individuals with high levels of leaf trichomes. Even when no evidence of a trade-off between plant resistance and tolerance was found, it should not be excluded because the expression of tolerance and its correlation with resistance seems to be a function of the magnitude of damage by herbivores in this population.

## Supplemental Information

10.7717/peerj.789/supp-1 Supplemental Information 1 Table S1 Raw data of vigor, reproductive and defensive traits of *Datura stramonium* plants. “Fam” indicates the number of genotype, “Ind” indicates individual plants”, “Treat” indicates the treatment application (insecticide), “0” untreated plants and “1” indicates plants treated with insecticide. “Rel damage” indicates the leaf damage loss due to herbivory weighted by total leaf area. “Resist” indicates the resistance level (1- relative leaf damage). “wi” indicates the relative fitness (the individual value between the population mean). The asterisks denote the genotypes that received the insecticide application employed to evaluate the cost of defense.
