# Supplementary material for: Evolution of resistance and tolerance to herbivores: testing the trade-off hypothesis
Source: PeerJ. 2015 Mar 3;3:e789. doi: 10.7717/peerj.789 (PMC4358663; doi:10.7717/peerj.789)
Supplement: Supplemental Information 1 — Raw data of vigor, reproductive and defensive traits of Datura stramonium plants. “Fam” indicates the number of genotype, “Ind” indicates individual plants”, “Treat” indicates the treatment application (insecticide), “0” untreated plants and “1” indicates plants treated with insecticide. “Rel damage” indicates the leaf damage loss due to herbivory weighted by total leaf area. “Resist” indicates the resistance level (1- relative leaf damage). “wi” indicates the relative fitness (the individual value between the population mean). The asterisks denote the genotypes that received the insecticide application employed to evaluate the cost of defense. [file peerj-03-789-s001.docx]

SUPPLEMENTAL MATERIAL OF “**Evolution of resistance and tolerance to herbivores: testing the trade-off hypothesis”**

| **Table S1**. Raw data of vigor, reproductive and defensive traits of *Datura stramonium* plants. “Fam” indicates the number of genotype, “Ind” indicates individual plants”, “Treat” indicates the treatment application (insecticide), “0” untreated plants and “1” indicates plants treated with insecticide. “Rel damage” indicates the leaf damage loss due to herbivory weighted by total leaf area. “Resist” indicates the resistance level (1- relative leaf damage). “wi” indicates the relative fitness (the individual value between the population mean). The asterisks denote the genotypes that received the insecticide application employed to evaluate the cost of defense. | | | | | | | | | | | | | | | | |
| --- | --- | --- | --- | --- | --- | --- | --- | --- | --- | --- | --- | --- | --- | --- | --- | --- |
| **Block** | **Fam** | **Ind** | **Treat** | **High (mm)** | **Branch (#)** | **Stem (mm)** | **Flower (#)** | **Fruit (#)** | **Seed (#)** | **Leaf area (cm^2^)** | **Leaf**  **damage** | **Rel damage** | **Resist** | **Leaf trichom** | **wi-fruit** | **wi-seed** |
| 1 | 1* | 2 | 0 | 164.2 | 3 | 2.9 | 5 | 1 | 58 | 3.694 | 0.26 | 0.056 | 0.943 | 17 | 0.166 | 0.043 |
| 1 | 1* | 6 | 0 | 280 | 5 | 5.87 | 3 | 4 | 498 | 1.537 | 0.02 | 0.008 | 0.988 | 18 | 0.663 | 0.369 |
| 2 | 1* | 7 | 0 | 175.3 | 3 | 4.65 | 5 | 1 | 59 | 1.116 | 0.002 | 0.001 | 0.998 | 51.4 | 0.166 | 0.044 |
| 2 | 1* | 11 | 0 | 164.7 | 2 | 3.7 | 1 | 1 | 1 | 0.97 | 0.11 | 0.095 | 0.904 | 29.333 | 0.166 | 0.0008 |
| 3 | 1* | 13 | 0 | 145.7 | 3 | 2.6 | 5 |  |  | 1.016 | 0.076 | 0.056 | 0.943 | 18.2 | 0 | 0 |
| 1 | 1* | 1 | 1 | 150.5 | 3 | 2.3 | 2 |  |  | 3.154 | 0.088 | 0.02 | 0.979 | 43.4 | 0 | 0 |
| 2 | 1* | 8 | 1 | 613.4 | 10 | 8.9 | 13 | 11 | 2644 | 3.608 | 0.002 | 0.0009 | 0.999 | 30.1 | 1.823 | 1.954 |
| 2 | 1* | 12 | 1 | 186.9 | 3 | 3.5 |  | 2 | 661 | 1.101 | 0.0162 | 0.007 | 0.992 | 13.25 | 0.332 | 0.489 |
| 3 | 1* | 14 | 1 | 319.7 | 5 | 4.45 |  | 2 | 293 | 1.836 | 0 | 0 | 1 | 58.3 | 0.332 | 0.217 |
| 3 | 1* | 16 | 1 | 210 | 3 | 6.7 | 5 | 3 | 294 | 4.241 | 0.331 | 0.058 | 0.941 | 53.9 | 0.497 | 0.218 |
| 1 | 2 | 3 | 0 | 289.4 | 9 | 6.26 | 13 | 7 | 1161 | 6.227 | 0.474 | 0.023 | 0.976 | 22.5 | 1.16 | 0.858 |
| 1 | 2 | 5 | 0 | 350.8 | 9 | 7.4 | 6 | 3 | 943 | 2.097 | 0.058 | 0.014 | 0.985 | 21.3 | 0.497 | 0.697 |
| 1 | 2 | 6 | 0 | 198.9 | 7 | 4.2 | 5 | 1 | 56 | 3.772 | 0.02 | 0.002 | 0.997 | 20.5 | 0.166 | 0.042 |
| 2 | 2 | 12 | 0 | 180.8 | 4 | 5.7 | 4 | 1 | 336 | 2.087 | 0.063 | 0.024 | 0.975 | 13.4 | 0.166 | 0.249 |
| 3 | 2 | 14 | 0 | 189.3 | 4 | 7.47 | 2 | 2 | 115 | 0.811 | 0.0065 | 0.003 | 0.996 | 33.3 | 0.332 | 0.085 |
| 3 | 2 | 15 | 0 | 377.9 | 8 | 7.89 | 7 | 6 | 613 | 1.489 | 0.017 | 0.015 | 0.984 | 38.9 | 0.994 | 0.454 |
| 1 | 3* | 2 | 0 | 456.1 | 8 | 9.13 | 13 | 9 | 2003 | 3.191 | 0.085 | 0.015 | 0.984 | 30.1 | 1.491 | 1.481 |
| 1 | 3* | 5 | 0 | 109 | 2 | 3.1 | 2 | 1 | 92 | 3.658 | 0.114 | 0.042 | 0.957 | 25.778 | 0.166 | 0.068 |
| 2 | 3* | 7 | 0 | 290.6 | 5 | 6.9 | 6 | 3 | 399 | 2.935 | 0.105 | 0.052 | 0.947 | 17.8 | 0.497 | 0.295 |
| 2 | 3* | 11 | 0 | 400 | 13 | 8.3 | 10 | 11 | 1753 | 3.227 | 0.006 | 0.001 | 0.998 | 24.8 | 1.822 | 1.296 |
| 1 | 3* | 3 | 1 | 322 | 7 | 7.4 | 9 | 5 | 540 | 1.762 | 0.142 | 0.038 | 0.961 | 38.3 | 0.828 | 0.4 |
| 2 | 3* | 10 | 1 | 157.9 | 3 | 3.4 | 4 | 1 | 114 | 3.368 | 0.097 | 0.034 | 0.965 | 43.2 | 0.166 | 0.085 |
| 3 | 3* | 15 | 1 | 130.8 | 3 | 5.64 | 4 | 3 | 340 | 2.192 | 0.01 | 0.004 | 0.995 | 48 | 0.497 | 0.252 |
| 3 | 3* | 17 | 1 | 166.7 | 6 | 4.89 | 3 | 5 | 516 | 5.415 | 0.0115 | 0.002 | 0.997 | 34 | 0.828 | 0.382 |
| 1 | 4 | 10 | 0 | 145.88 | 2 | 2.7 |  | 1 | 24 | 7.066 | 0.017 | 0.001 | 0.998 | 25 | 0.166 | 0.018 |
| 2 | 4 | 12 | 0 | 298.5 | 4 | 4.6 | 5 | 3 | 303 | 2.307 | 0.078 | 0.014 | 0.985 | 14.778 | 0.497 | 0.224 |
| 2 | 4 | 13 | 0 | 278.9 | 7 | 4.45 | 4 | 3 | 209 | 3.633 | 0.047 | 0.016 | 0.983 | 21.8 | 0.497 | 0.155 |
| 3 | 4 | 16 | 0 | 176.9 | 3 | 3.3 | 2 | 2 | 25 | 1.758 | 0.04 | 0.016 | 0.983 | 36.286 | 0.332 | 0.019 |
| 2 | 5* | 7 | 0 | 88 | 2 | 3.5 | 1 | 1 | 91 | 6.126 | 0.758 | 0.105 | 0.894 | 22.4 | 0.166 | 0.068 |
| 3 | 5* | 16 | 0 | 238.9 | 2 | 5.3 | 2 | 3 | 184 | 5.678 | 0.202 | 0.016 | 0.983 | 22 | 0.497 | 0.136 |
| 1 | 5* | 5 | 1 | 166.9 | 3 | 4.2 | 2 | 2 | 130 | 4.39 | 0.178 | 0.034 | 0.966 | 23.308 | 0.332 | 0.097 |
| 2 | 5* | 10 | 1 | 220 | 4 | 3.56 | 6 | 1 | 105 | 0.586 | 0 | 0 | 1 | 16.667 | 0.166 | 0.078 |
| 2 | 5* | 11 | 1 | 111.9 | 3 | 4.6 | 2 | 1 | 147 | 1.344 | 0 | 0 | 1 | 19.333 | 0.166 | 0.109 |
| 3 | 5* | 17 | 1 | 167.9 | 4 | 3.57 |  | 3 | 313 | 5.177 | 1.682 | 0.160 | 0.839 | 27.5 | 0.497 | 0.232 |
| 1 | 6* | 6 | 0 | 200 | 4 | 4.52 | 5 | 3 | 235 | 4.451 | 0.253 | 0.042 | 0.957 | 27.5 | 0.497 | 0.174 |
| 2 | 6* | 11 | 0 | 146.5 | 3 | 2.87 | 3 | 1 | 78 | 1.478 | 0.017 | 0.007 | 0.992 | 18.9 | 0.166 | 0.058 |
| 3 | 6* | 14 | 0 | 226.8 | 7 | 5.97 | 10 | 5 | 667 | 8.258 | 0.295 | 0.021 | 0.979 | 27.1 | 0.829 | 0.493 |
| 3 | 6* | 18 | 0 | 120.8 | 3 | 1.98 | 2 |  |  | 0.834 | 0.123 | 0.162 | 0.837 | 15.143 | 0 | 0 |
| 1 | 6* | 1 | 1 | 180 | 5 | 5.3 | 7 | 5 | 1156 | 7.513 | 0.024 | 0.001 | 0.999 | 23.6 | 0.829 | 0.855 |
| 2 | 6* | 12 | 1 | 98.4 | 2 | 2.37 | 2 |  |  | 0.538 | 0 | 0 | 1 | 9 | 0 | 0 |
| 3 | 6* | 13 | 1 | 217.6 | 10 | 6.67 | 8 | 4 | 925 | 9.989 | 0.513 | 0.05 | 0.95 | 25.4 | 0.663 | 0.684 |
| 3 | 6* | 15 | 1 | 100.5 | 1 | 2.45 | 2 |  |  | 8.771 | 0.196 | 0.017 | 0.983 | 42.4 | 0 | 0 |
| 1 | 7 | 3 | 0 | 198.8 | 5 | 4.7 | 3 | 3 | 530 | 4.499 | 0.018 | 0.001 | 0.999 | 32.7 | 0.497 | 0.392 |
| 1 | 7 | 5 | 0 | 109.7 | 3 | 4.89 | 4 | 1 | 57 | 1.608 | 0 | 0 | 1 | 21.333 | 0.166 | 0.043 |
| 1 | 7 | 6 | 0 | 179.8 | 4 | 4.76 | 5 | 2 | 147 | 5.72 | 0.584 | 0.227 | 0.773 | 26 | 0.332 | 0.109 |
| 3 | 7 | 15 | 0 | 150 | 4 | 5.78 | 3 | 1 | 863 | 5.814 | 0.065 | 0.014 | 0.986 | 33.5 | 0.166 | 0.638 |
| 3 | 7 | 16 | 0 | 112.8 | 3 | 5.8 | 2 | 1 | 27 | 4.907 | 0.52 | 0.059 | 0.948 | 25.438 | 0.166 | 0.02 |
| 3 | 7 | 17 | 0 | 130.5 | 6 | 4.89 | 3 | 3 | 414 | 6.542 | 0.832 | 0.094 | 0.906 | 17.9 | 0.497 | 0.306 |
| 1 | 8* | 6 | 0 | 189 | 2 | 3.1 | 2 | 1 | 101 | 7.427 | 0.097 | 0.010 | 0.99 | 20.9 | 0.166 | 0.075 |
| 2 | 8* | 10 | 0 | 144.7 | 4 | 3.7 | 5 | 1 | 178 | 1.046 | 0.013 | 0.012 | 0.988 | 19.75 | 0.166 | 0.132 |
| 3 | 8* | 13 | 0 | 310 | 5 | 6.8 | 3 | 2 | 202 | 3.925 | 0.011 | 0.002 | 0.998 | 24.1 | 0.332 | 0.15 |
| 3 | 8* | 14 | 0 | 398.5 | 10 | 10.92 | 8 | 14 | 4681 | 10.952 | 0.187 | 0.014 | 0.986 | 20.1 | 2.312 | 3.46 |
| 1 | 8* | 2 | 1 | 223.9 | 4 | 8.4 | 3 | 2 | 205 | 5.113 | 0.006 | 0.0007 | 0.9993 | 42.25 | 0.332 | 0.152 |
| 2 | 8* | 9 | 1 | 300.9 | 5 | 5.4 | 4 | 3 | 204 | 8.135 | 0.544 | 0.065 | 0.935 | 35 | 0.497 | 0.151 |
| 2 | 8* | 12 | 1 | 310.8 | 4 | 5.87 | 2 | 2 | 130 | 2.256 | 0 | 0 | 1 | 29.3 | 0.332 | 0.097 |
| 3 | 8* | 15 | 1 | 256.9 | 7 | 9.3 | 6 | 6 | 1643 | 7.676 | 1.023 | 0.099 | 0.901 | 33.1 | 0.994 | 1.215 |
| 3 | 8* | 17 | 1 | 522 | 14 | 12.25 | 19 | 23 | 5483 | 8.614 | 0.067 | 0.006 | 0.994 | 44.5 | 3.89 | 4.052 |
| 1 | 9* | 1 | 0 | 144.7 | 3 | 3.3 | 2 | 1 | 125 | 7.728 | 0.24 | 0.047 | 0.953 | 31 | 0.166 | 0.093 |
| 2 | 9* | 8 | 0 | 300.76 | 3 | 6.8 | 3 | 3 | 302 | 3.536 | 0.064 | 0.014 | 0.986 | 28.858 | 0.497 | 0.224 |
| 2 | 9* | 12 | 0 | 234.5 | 3 | 6.87 | 6 | 2 | 142 | 1.928 | 0.021 | 0.018 | 0.982 | 23.778 | 0.332 | 0.11 |
| 3 | 9* | 13 | 0 | 315.7 | 9 | 6.28 | 7 | 5 | 355 | 9.859 | 0.16 | 0.023 | 0.977 | 34.4 | 0.829 | 0.263 |
| 1 | 9* | 2 | 1 | 210.8 | 5 | 7.8 | 2 | 3 | 235 | 3.633 | 0.164 | 0.016 | 0.984 | 33.833 | 0.497 | 0.174 |
| 2 | 9* | 9 | 1 | 485.9 | 15 | 9.27 | 16 | 11 | 1673 | 11.38 | 0.176 | 0.012 | 0.988 | 30.8 | 1.823 | 1.237 |
| 2 | 9* | 11 | 1 | 160.9 | 3 | 9.27 | 4 |  |  | 1.105 | 0 | 0 | 1 | 13.444 | 0 | 0 |
| 1 | 10 | 4 | 0 | 134.8 | 5 | 4.2 | 2 | 3 | 46 | 3.136 | 0.143 | 0.047 | 0.953 | 33.2 | 0.497 | 0.034 |
| 1 | 10 | 5 | 0 | 137.9 | 2 | 3.9 |  |  | 55 | 2.194 | 0.33 | 0.215 | 0.785 | 12.572 | 0 | 0.041 |
| 3 | 10 | 16 | 0 | 177.2 | 3 | 3.81 | 1 | 3 | 282 | 3.237 | 0.126 | 0.035 | 0.965 | 47.8 | 0.497 | 0.21 |
| 1 | 11* | 3 | 0 | 176 | 4 | 3.8 | 7 | 2 | 68 | 2.381 | 0.03 | 0.004 | 0.995 | 18.25 | 0.332 | 0.051 |
| 2 | 11* | 8 | 0 | 134.8 | 2 | 4.6 | 5 | 1 | 34 | 3.885 | 0.098 | 0.023 | 0.976 | 30 | 0.166 | 0.026 |
| 2 | 11* | 12 | 0 | 278 | 7 | 5.6 | 10 | 2 | 422 | 4.402 | 0.080 | 0.014 | 0.985 | 36.7 | 0.332 | 0.312 |
| 3 | 11* | 15 | 0 | 176.9 | 3 | 4.6 | 3 | 2 | 67 | 5.286 | 0.207 | 0.029 | 0.97 | 39.222 | 0.332 | 0.05 |
| 3 | 11* | 17 | 0 | 276.9 | 11 | 5.65 | 9 | 5 | 1056 | 4.77 | 0.068 | 0.009 | 0.99 | 40.8 | 0.829 | 0.781 |
| 2 | 11* | 11 | 1 | 200 | 2 | 4 | 3 | 1 | 212 | 6.13 | 0.006 | 0.001 | 0.998 | 43.2 | 0.166 | 0.157 |
| 3 | 11* | 14 | 1 | 160.5 | 4 | 4.76 | 3 | 1 | 35 | 1.588 | 0.06 | 0.032 | 0.967 | 62.4 | 0.166 | 0.026 |
| 3 | 11* | 16 | 1 | 124 | 1 | 2.7 |  | 1 | 36 | 1.642 | 0.085 | 0.063 | 0.936 | 15.75 | 0.166 | 0.027 |
| 2 | 12 | 7 | 0 | 327.8 | 5 | 7.8 | 7 | 3 | 512 | 3.688 | 0.116 | 0.036 | 0.964 | 36 | 0.497 | 0.379 |
| 2 | 12 | 10 | 0 | 407.7 | 8 | 6.24 | 10 | 5 | 554 | 2.46 | 0.003 | 0.001 | 0.999 | 45.3 | 0.829 | 0.41 |
| 3 | 12 | 15 | 0 | 155.5 | 5 | 4.07 | 4 | 3 | 264 | 3.802 | 0.207 | 0.049 | 0.951 | 27.143 | 0.497 | 0.196 |
| 1 | 13 | 1 | 0 | 200 | 6 | 4.86 | 4 | 3 | 551 | 6.345 | 0.008 | 0.0008 | 0.9992 | 27.3 | 0.497 | 0.41 |
| 3 | 13 | 13 | 0 | 178.97 | 8 | 5.6 | 5 | 4 | 471 | 5.189 | 0.123 | 0.02 | 0.98 | 37.2 | 0.663 | 0.349 |
| 3 | 13 | 17 | 0 | 240.3 | 8 | 7 | 8 | 5 | 825 | 5.494 | 0.701 | 0.045 | 0.955 | 49.4 | 0.829 | 0.61 |
| 1 | 14* | 3 | 0 | 156.8 | 2 | 2.15 |  | 1 | 34 | 1.56 | 0.026 | 0.015 | 0.985 | 14.9 | 0.166 | 0.026 |
| 2 | 14* | 7 | 0 | 217.87 | 5 | 6.2 | 4 | 3 | 329 | 1.268 | 0.018 | 0.006 | 0.994 | 27.2 | 0.497 | 0.244 |
| 2 | 14* | 9 | 0 | 134.7 | 3 | 3.9 | 2 | 1 | 80 | 2.274 | 0.035 | 0.012 | 0.988 | 52 | 0.166 | 0.06 |
| 3 | 14* | 15 | 0 | 398.5 | 17 | 9.59 | 23 | 9 | 2556 | 21.033 | 0.456 | 0.018 | 0.982 | 21.222 | 1.491 | 1.889 |
| 1 | 14* | 1 | 1 | 276.56 | 6 | 7.2 | 5 | 7 | 888 | 4.968 | 0.173 | 0.018 | 0.982 | 33.5 | 1.16 | 0.657 |
| 1 | 14* | 4 | 1 | 150 | 2 | 3.9 | 1 | 1 | 110 | 0.645 | 0 | 0 | 1 | 49 | 0.166 | 0.082 |
| 2 | 14* | 8 | 1 | 178.9 | 4 | 4.6 | 6 | 1 | 403 | 10.981 | 0.076 | 0.007 | 0.993 | 44.6 | 0.166 | 0.298 |
| 3 | 14* | 13 | 1 | 520.76 | 17 | 10.5 | 8 | 17 | 5227 | 16.941 | 0.118 | 0.007 | 0.993 | 40.1 | 2.816 | 3.863 |
| 3 | 14* | 17 | 1 | 213.6 | 6 | 6.5 | 7 | 6 | 2413 | 5.02 | 0.589 | 0.067 | 0.933 | 45.8 | 0.994 | 1.784 |
| 1 | 15* | 2 | 0 | 128.3 | 4 | 2.7 | 7 | 1 | 6 | 6.918 | 0.214 | 0.034 | 0.966 | 26 | 0.166 | 0.005 |
| 2 | 15* | 7 | 0 | 153.5 | 4 | 3.79 | 3 | 2 | 227 | 2.801 | 0.13 | 0.039 | 0.961 | 75 | 0.332 | 0.168 |
| 2 | 15* | 13 | 0 | 389 | 9 | 7.8 | 16 | 18 | 5843 | 6.334 | 0.088 | 0.011 | 0.989 | 41.455 | 2.982 | 4.318 |
| 3 | 15* | 15 | 0 | 235.7 | 4 | 4.9 | 3 | 5 | 734 | 5.345 | 0.066 | 0.011 | 0.989 | 30.7 | 0.829 | 0.543 |
| 3 | 15* | 16 | 0 | 211.4 | 3 | 3.29 |  | 4 | 454 | 5.205 | 0.6 | 0.181 | 0.819 | 23 | 0.663 | 0.336 |
| 1 | 15* | 1 | 1 | 128.9 | 3 | 2.74 | 6 | 1 | 148 | 1.358 | 0.025 | 0.01 | 0.99 | 11 | 0.166 | 0.11 |
| 1 | 15* | 3 | 1 | 215.6 | 4 | 3.5 | 6 | 0 |  | 10.542 | 0.081 | 0.005 | 0.995 | 48.1 | 0 | 0 |
| 3 | 15* | 17 | 1 | 338.5 | 17 | 9.14 | 13 | 10 | 2037 | 9.077 | 0.537 | 0.053 | 0.947 | 30.8 | 1.657 | 1.51 |
| 3 | 15* | 18 | 1 | 610 | 28 | 11.73 | 22 | 23 | 8366 | 12.013 | 0.114 | 0.009 | 0.991 | 27.5 | 3.81 | 6.183 |
| 1 | 16* | 2 | 0 | 349.5 | 9 | 8.8 | 8 | 10 | 319 | 2.404 | 0.069 | 0.021 | 0.979 | 52 | 1.657 | 0.236 |
| 1 | 16* | 4 | 0 | 160.5 | 3 | 4.65 | 1 | 1 | 14 | 2.37 | 0.0008 | 0.0001 | 0.999 | 19.2 | 0.166 | 0.011 |
| 1 | 16* | 6 | 0 | 105.3 | 2 | 3 | 3 | 2 | 46 | 2.12 | 0.061 | 0.0194 | 0.981 | 31.572 | 0.332 | 0.034 |
| 2 | 16* | 7 | 0 | 398.74 | 9 | 7.5 | 5 | 6 | 1038 | 6.222 | 0.152 | 0.018 | 0.982 | 21.5 | 0.994 | 0.768 |
| 3 | 16* | 15 | 0 | 548.5 | 19 | 14.5 | 23 | 23 | 8366 | 13.185 | 0.168 | 0.01 | 0.99 | 39.2 | 3.81 | 6.183 |
| 3 | 16* | 17 | 0 | 326.7 | 9 | 6.57 | 15 | 3 | 249 | 8.001 | 0.45 | 0.034 | 0.966 | 33.3 | 0.497 | 0.185 |
| 1 | 16* | 1 | 1 | 227.9 | 4 | 5.5 | 5 | 2 | 309 | 4.056 | 0.201 | 0.026 | 0.974 | 30.8 | 0.332 | 0.229 |
| 1 | 16* | 5 | 1 | 112.4 | 3 | 2.89 | 2 | 1 | 90 | 2.561 | 0.064 | 0.023 | 0.977 | 29.8 | 0.166 | 0.067 |
| 2 | 16* | 8 | 1 | 168.9 | 1 | 3.12 | 1 | 1 | 5 | 0.898 | 0.08 | 0.092 | 0.908 | 24.858 | 0.166 | 0.004 |
| 2 | 16* | 11 | 1 | 315.9 | 5 | 5.6 | 5 | 2 | 513 | 3.016 | 0.041 | 0.005 | 0.995 | 16.7 | 0.332 | 0.38 |
| 3 | 16* | 16 | 1 | 557.9 | 28 | 15.6 | 31 | 36 | 8490 | 15.78 | 0.095 | 0.004 | 0.996 | 29.6 | 5.964 | 6.275 |
| 3 | 16* | 18 | 1 | 350.9 | 9 | 7.77 | 8 | 5 | 2825 | 5.696 | 0.0205 | 0.005 | 0.995 | 25 | 0.829 | 2.089 |
| 1 | 17* | 4 | 0 | 98.3 | 3 | 2.8 | 2 | 1 | 37 | 0.693 | 0 | 0 | 1 | 30.667 | 0.166 | 0.028 |
| 2 | 17* | 7 | 0 | 217.8 | 5 | 5.6 | 2 | 3 | 377 | 1.032 | 0 | 0 | 1 | 44.5 | 0.497 | 0.279 |
| 3 | 17* | 14 | 0 | 279.9 | 7 | 8.4 | 6 | 5 | 452 | 13.558 | 0.235 | 0.01 | 0.99 | 35.3 | 0.829 | 0.335 |
| 1 | 17* | 1 | 1 | 185.9 | 4 | 5.9 | 5 | 4 | 265 | 2.88 | 0.066 | 0.017 | 0.983 | 27 | 0.663 | 0.196 |
| 1 | 17* | 2 | 1 | 193.5 | 5 | 5 | 6 | 3 | 26 | 2.474 | 0.0085 | 0.003 | 0.997 | 23.7 | 0.497 | 0.02 |
| 2 | 17* | 8 | 1 | 367 | 6 | 6.3 | 5 | 6 | 1175 | 4.821 | 0.002 | 0.0003 | 0.9997 | 20.1 | 0.994 | 0.869 |
| 2 | 17* | 12 | 1 | 340.5 | 10 | 7.3 | 12 | 7 | 1950 | 3.144 | 0.062 | 0.0178 | 0.982 | 30.5 | 1.156 | 1.442 |
| 1 | 18 | 5 | 0 | 186.34 | 2 | 2.9 | 1 | 1 | 274 | 0.245 | 0 | 0 | 1 | 10.5 | 0.166 | 0.203 |
| 1 | 18 | 6 | 0 | 130 | 4 | 3.37 | 4 | 2 | 472 | 1.92 | 0 | 0 | 1 | 55 | 0.332 | 0.349 |
| 2 | 18 | 12 | 0 | 345.65 | 8 | 7.31 | 10 | 7 | 1420 | 8.248 | 0.039 | 0.004 | 0.996 | 56.3 | 1.156 | 1.05 |
| 3 | 18 | 17 | 0 | 513.8 | 23 | 12.73 | 19 | 22 | 5792 | 15.678 | 0.311 | 0.013 | 0.987 | 47.6 | 3.645 | 4.281 |
| 1 | 19* | 1 | 0 | 395.9 | 8 | 6.65 | 8 | 4 | 682 | 11.463 | 0.036 | 0.003 | 0.997 | 19.7 | 0.663 | 0.504 |
| 1 | 19* | 5 | 0 | 412.7 | 8 | 10.7 | 6 | 7 | 1195 | 2.1 | 0.085 | 0.089 | 0.911 | 86.167 | 1.16 | 0.884 |
| 2 | 19* | 11 | 0 | 231.2 | 5 | 4.58 | 3 | 3 | 137 | 2.824 | 0.153 | 0.029 | 0.971 | 30.7 | 0.497 | 0.102 |
| 3 | 19* | 15 | 0 | 215.8 | 8 | 4.23 | 2 | 5 | 584 | 5.737 | 0.422 | 0.031 | 0.969 | 37.7 | 0.829 | 0.432 |
| 1 | 19* | 3 | 1 | 186.7 | 1 | 2.22 | 3 | 1 | 100 | 10.655 | 0.0315 | 0.003 | 0.997 | 46.9 | 0.166 | 0.074 |
| 2 | 19* | 12 | 1 | 320.3 | 12 | 6.4 | 4 | 5 | 1800 | 10.852 | 0.162 | 0.018 | 0.982 | 45.3 | 0.823 | 1.331 |
| 3 | 19* | 16 | 1 | 548.9 | 64 | 13.36 | 13 | 36 | 11547 | 14.278 | 0.166 | 0.006 | 0.994 | 45.9 | 5.964 | 8.534 |
| 3 | 19* | 18 | 1 | 489.1 | 12 | 8.9 | 5 | 10 | 1388 | 6.884 | 0.01 | 0.001 | 0.999 | 47 | 1.657 | 1.026 |
| 1 | 20* | 1 | 0 | 176.98 | 3 | 2.3 | 1 | 1 | 19 | 6.945 | 0.063 | 0.008 | 0.992 | 36.1 | 0.166 | 0.015 |
| 1 | 20* | 8 | 0 | 234.7 | 4 | 4.41 | 5 | 2 | 37 | 3.724 | 0.127 | 0.05 | 0.95 | 19.9 | 0.332 | 0.028 |
| 2 | 20* | 12 | 0 | 135.76 | 2 | 3.4 | 4 | 1 | 112 | 9.403 | 0.069 | 0.005 | 0.995 | 42.8 | 0.166 | 0.083 |
| 3 | 20* | 16 | 0 | 478.5 | 21 | 8.76 | 25 | 9 | 2344 | 9.723 | 0 | 0 | 1 | 58 | 1.491 | 1.733 |
| 3 | 20* | 18 | 0 | 305.4 | 14 | 7.26 | 12 | 10 | 2051 | 10.491 | 0.163 | 0.012 | 0.988 | 35 | 1.657 | 1.516 |
| 1 | 20* | 3 | 1 | 256 | 6 | 7.1 | 5 | 4 | 518 | 5.151 | 0.506 | 0.045 | 0.955 | 13.1 | 0.663 | 0.383 |
| 2 | 20* | 11 | 1 | 156.8 | 3 | 3.9 | 2 | 1 | 8 | 1.741 | 0.0145 | 0.003 | 0.997 | 19.7 | 0.166 | 0.006 |
| 3 | 20* | 15 | 1 | 365.21 | 14 | 9 | 10 | 13 | 3403 | 7.534 | 0.635 | 0.045 | 0.955 | 25.8 | 2.154 | 2.515 |
| 3 | 20* | 17 | 1 | 210 | 5 | 5.1 | 3 | 4 | 338 | 1.966 | 0.029 | 0.003 | 0.997 | 40.5 | 0.663 | 0.25 |
| 2 | 21 | 3 | 0 | 321.7 | 4 | 5.7 | 2 | 3 | 939 | 8.587 | 0.272 | 0.022 | 0.977 | 30.1 | 0.497 | 0.694 |
| 1 | 21 | 9 | 0 | 334.7 | 4 | 4.7 | 5 |  |  | 10.708 | 0.379 | 0.022 | 0.977 | 22.7 | 0 | 0 |
| 2 | 21 | 13 | 0 | 467.89 | 9 | 9.5 | 10 | 6 | 1198 | 4.262 | 0.07 | 0.008 | 0.991 | 43.8 | 0.994 | 0.886 |
| 3 | 21 | 14 | 0 | 367.9 | 13 | 7.5 | 16 | 14 | 3448 | 13.137 | 0.49 | 0.021 | 0.978 | 18 | 2.32 | 2.549 |
| 3 | 21 | 15 | 0 | 150.4 | 3 | 3.84 | 2 | 1 | 313 | 5.553 | 0.055 | 0.007 | 0.992 | 32.8 | 0.166 | 0.232 |
| 3 | 21 | 17 | 0 | 400 | 48 | 13.47 | 28 | 34 | 10952 | 20.537 | 0.085 | 0.005 | 0.994 | 23.7 | 5.632 | 8.094 |
| 1 | 22* | 5 | 0 | 245.9 | 5 | 6.4 | 6 | 3 | 159 | 5.319 | 0.035 | 0.008 | 0.992 | 38.1 | 0.497 | 0.118 |
| 2 | 22* | 11 | 0 | 170.4 | 2 | 4.2 | 1 | 1 | 52 | 3.022 | 0.01 | 0.004 | 0.996 | 38 | 0.166 | 0.039 |
| 3 | 22* | 13 | 0 | 449.7 | 14 | 9.98 | 12 | 15 | 1964 | 11.164 | 0.492 | 0.032 | 0.968 | 35.1 | 2.485 | 1.452 |
| 3 | 22* | 17 | 0 | 500.23 | 11 | 7.3 | 9 | 10 | 1801 | 11.398 | 0.352 | 0.032 | 0.968 | 28 | 1.657 | 1.331 |
| 1 | 22* | 1 | 1 | 338.9 | 9 | 6.5 | 10 | 7 | 1370 | 6.6475 | 0.005 | 0.001 | 0.999 | 30.5 | 1.16 | 1.013 |
| 1 | 22* | 4 | 1 | 210.3 | 6 | 8.8 | 7 | 2 | 391 | 2.6272 | 0.24 | 0.061 | 0.939 | 47.8 | 0.332 | 0.289 |
| 2 | 22* | 10 | 1 | 387.9 | 4 | 5.6 | 5 | 2 | 253 | 1.603 | 0 | 0 | 1 | 24.3 | 0.332 | 0.187 |
| 3 | 22* | 18 | 1 | 498.5 | 13 | 10.93 | 15 | 19 | 3783 | 19.391 | 0.103 | 0.003 | 0.997 | 18 | 3.148 | 2.796 |
| 3 | 23 | 1 | 0 | 800 | 28 | 18.5 | 37 | 34 | 11716 | 17.87 | 1.311 | 0.053 | 0.947 | 37.6 | 5.632 | 8.659 |
| 1 | 23 | 2 | 0 | 178.43 | 4 | 4.2 | 2 | 2 | 57 | 4.607 | 0.13 | 0.025 | 0.975 | 47.572 | 0.332 | 0.043 |
| 1 | 23 | 4 | 0 | 198.5 | 3 | 4.56 | 1 | 2 | 175 | 3.277 | 0.118 | 0.015 | 0.985 | 21.667 | 0.332 | 0.13 |
| 1 | 23 | 9 | 0 | 145 | 2 | 3.7 | 2 | 1 | 31 | 1.135 | 0 | 0 | 1 | 68.5 | 0.166 | 0.023 |
| 2 | 23 | 15 | 0 | 102.4 | 3 | 2.43 | 1 | 1 | 43 | 2.492 | 0.0162 | 0.005 | 0.995 | 38.625 | 0.166 | 0.032 |
| 3 | 23 | 16 | 0 | 157.9 | 2 | 2.7 | 1 | 1 | 14 | 4.896 | 0.062 | 0.018 | 0.982 | 21.4 | 0.166 | 0.011 |
| 3 | 23 | 17 | 0 | 605.7 | 34 | 13.06 | 22 | 10 | 1720 | 18.871 | 0.209 | 0.012 | 0.988 | 36.8 | 1.657 | 1.272 |
| 3 | 24 | 7 | 0 | 559.7 | 8 | 9.1 | 16 | 8 | 1662 | 6.848 | 0.071 | 0.008 | 0.992 | 41 | 1.326 | 1.229 |
| 2 | 24 | 11 | 0 | 178.9 | 4 | 3.2 | 4 | 1 | 78 | 2.07 | 0.028 | 0.012 | 0.988 | 12 | 0.166 | 0.058 |
| 2 | 24 | 15 | 0 | 466.4 | 17 | 11.64 | 14 | 8 | 1104 | 11.628 | 0.039 | 0.002 | 0.998 | 51 | 1.326 | 0.816 |
| 3 | 24 | 16 | 0 | 398.59 | 10 | 7.8 | 17 | 7 | 1002 | 4.227 | 0.235 | 0.047 | 0.953 | 69.9 | 1.16 | 0.741 |
| 3 | 24 | 17 | 0 | 347.9 | 13 | 8.3 | 11 | 15 | 944 | 12.403 | 0.208 | 0.017 | 0.983 | 30.6 | 2.485 | 0.698 |
| 3 | 24 | 18 | 0 | 349.21 | 10 | 7.96 | 9 | 12 | 3899 | 14.638 | 0.709 | 0.025 | 0.975 | 27 | 1.988 | 2.882 |
| 2 | 25 | 11 | 0 | 246.5 | 5 | 6.7 | 10 |  |  | 4.318 | 0.691 | 0.11 | 0.89 | 26.7 | 0 | 0 |
| 3 | 25 | 13 | 0 | 489.67 | 21 | 10.6 | 25 | 18 | 5179 | 16.412 | 0.038 | 0.002 | 0.993 | 22.1 | 2.982 | 3.828 |
| 3 | 25 | 15 | 0 | 356.7 | 10 | 8.5 | 14 | 3 | 1094 | 7.49 | 0.067 | 0.009 | 0.991 | 50.2 | 0.497 | 0.809 |
| 3 | 25 | 16 | 0 | 326.8 | 7 | 5.9 | 10 | 4 | 824 | 10.128 | 0.128 | 0.016 | 0.984 | 75.9 | 0.663 | 0.609 |
| 1 | 26* | 1 | 0 | 650 | 26 | 11.6 | 18 | 27 | 6929 | 20.241 | 0.029 | 0.001 | 0.999 | 21.3 | 4.473 | 5.121 |
| 3 | 26* | 14 | 0 | 500 | 30 | 11.58 | 18 | 22 | 2306 | 10.477 | 0.971 | 0.045 | 0.955 | 29.3 | 3.645 | 1.705 |
| 3 | 26* | 16 | 0 | 418.54 | 16 | 8.96 | 20 | 7 | 2095 | 17.493 | 0.313 | 0.018 | 0.982 | 23.2 | 1.16 | 1.549 |
| 3 | 26* | 17 | 0 | 345.76 | 18 | 8.94 | 15 | 14 | 3073 | 14.712 | 0.604 | 0.043 | 0.957 | 29.5 | 2.32 | 2.271 |
| 1 | 26* | 5 | 1 | 125.98 | 13 | 2.9 | 17 |  |  | 10.131 | 0.207 | 0.014 | 0.986 | 28.9 | 0 | 0 |
| 1 | 26* | 6 | 1 | 186.87 | 6 | 4.98 | 5 | 3 | 166 | 1.29 | 0.003 | 0.004 | 0.996 | 79.75 | 0.497 | 0.123 |
| 3 | 26* | 13 | 1 | 334.87 | 17 | 8.5 | 8 | 12 | 3313 | 9.866 | 0.327 | 0.024 | 0.976 | 33.7 | 1.988 | 2.449 |
| 3 | 26* | 18 | 1 | 206.6 | 4 | 5.78 | 2 | 1 | 464 | 4.076 | 0.014 | 0.003 | 0.997 | 30.333 | 0.166 | 0.343 |
| 2 | 26* | 12 | 1 | 165.7 | 2 | 3.2 |  | 1 | 195 | 7.108 | 0.028 | 0.003 | 0.997 | 31.7 | 0.166 | 0.145 |
| 1 | 27* | 2 | 0 | 606.9 | 17 | 14.66 | 19 | 13 | 5437 | 24.466 | 2.503 | 0.062 | 0.938 | 25.4 | 2.154 | 4.018 |
| 1 | 27* | 4 | 0 | 345.76 | 9 | 7.8 | 13 | 9 | 2855 | 4.068 | 0.049 | 0.02 | 0.98 | 41.9 | 1.491 | 2.11 |
| 2 | 27* | 7 | 0 | 89.23 | 1 | 2.7 |  | 1 | 189 | 14.254 | 0.876 | 0.052 | 0.948 | 31.3 | 0.166 | 0.14 |
| 3 | 27* | 13 | 0 | 470 | 19 | 10.29 | 31 | 1 | 3808 | 14.262 | 0.911 | 0.055 | 0.945 | 29.4 | 0.166 | 2.815 |
| 3 | 27* | 14 | 0 | 637.9 | 160 | 14.8 | 26 | 18 | 4390 | 21.131 | 0.246 | 0.013 | 0.987 | 27.4 | 2.982 | 3.245 |
| 1 | 27* | 3 | 1 | 332.87 | 8 | 4.94 | 16 | 5 | 692 | 5.672 | 0.367 | 0.026 | 0.974 | 19 | 0.829 | 0.512 |
| 1 | 27* | 5 | 1 | 123 | 2 | 3.6 | 1 | 1 | 139 | 0.762 | 0 | 0 | 1 | 29.5 | 0.166 | 0.11 |
| 2 | 27* | 12 | 1 | 127.87 | 2 | 2.2 | 1 | 1 | 140 | 13.428 | 0.233 | 0.019 | 0.981 | 21.6 | 0.166 | 0.11 |
| 3 | 27* | 15 | 1 | 524.87 | 19 | 14.33 | 18 | 21 | 10823 | 19.938 | 0.562 | 0.026 | 0.974 | 14.3 | 3.479 | 7.999 |
| 3 | 28 | 5 | 0 | 90 | 1 | 3.8 |  | 1 | 81 | 4.274 | 0.02 | 0.002 | 0.998 | 49.3 | 0.166 | 0.06 |
| 1 | 28 | 11 | 0 | 157.98 | 1 | 3.9 | 2 | 1 | 310 | 8.306 | 0.082 | 0.006 | 0.994 | 80.2 | 0.166 | 0.23 |
| 2 | 28 | 14 | 0 | 600 | 52 | 16.19 | 37 | 43 | 14133 | 17.177 | 0.074 | 0.002 | 0.998 | 68.6 | 7.123 | 10.445 |
| 3 | 28 | 15 | 0 | 256.9 | 18 | 12.2 | 15 | 2 | 644 | 6.452 | 0.12 | 0.007 | 0.993 | 94.111 | 0.332 | 0.476 |
| 3 | 28 | 16 | 0 | 689.3 | 102 | 18.81 | 29 | 12 | 4233 | 26.902 | 0.044 | 0.001 | 0.999 | 47.4 | 1.988 | 3.129 |
| 3 | 28 | 17 | 0 | 386.9 | 12 | 12.3 | 10 | 9 | 2306 | 1.125 | 0 | 0 | 1 | 96.7 | 1.491 | 1.705 |
